# OncodriveCLUSTL: a sequence-based clustering method to identify cancer drivers

**DOI:** 10.1093/bioinformatics/btz501

**Published:** 2019-06-22

**Authors:** Claudia Arnedo-Pac, Loris Mularoni, Ferran Muiños, Abel Gonzalez-Perez, Nuria Lopez-Bigas

**Affiliations:** 1 Institute for Research in Biomedicine (IRB Barcelona), The Barcelona Institute of Science and Technology (BIST), Barcelona, Spain; 2 Research Program on Biomedical Informatics, Universitat Pompeu Fabra, Barcelona, Spain; 3 Institució Catalana de Recerca i Estudis Avançats (ICREA), Passeig Lluís Companys 23, Barcelona 08010, Spain

## Abstract

**Motivation:**

Identification of the genomic alterations driving tumorigenesis is one of the main goals in oncogenomics research. Given the evolutionary principles of cancer development, computational methods that detect signals of positive selection in the pattern of tumor mutations have been effectively applied in the search for cancer genes. One of these signals is the abnormal clustering of mutations, which has been shown to be complementary to other signals in the detection of driver genes.

**Results:**

We have developed OncodriveCLUSTL, a new sequence-based clustering algorithm to detect significant clustering signals across genomic regions. OncodriveCLUSTL is based on a local background model derived from the simulation of mutations accounting for the composition of tri- or penta-nucleotide context substitutions observed in the cohort under study. Our method can identify known clusters and *bona-fide* cancer drivers across cohorts of tumor whole-exomes, outperforming the existing OncodriveCLUST algorithm and complementing other methods based on different signals of positive selection. Our results indicate that OncodriveCLUSTL can be applied to the analysis of non-coding genomic elements and non-human mutations data.

**Availability and implementation:**

OncodriveCLUSTL is available as an installable Python 3.5 package. The source code and running examples are freely available at https://bitbucket.org/bbglab/oncodriveclustl under GNU Affero General Public License.

**Supplementary information:**

Supplementary data are available at *Bioinformatics* online.

## 1 Introduction

Identification of the alterations driving tumorigenesis is a major goal of cancer research. Knowledge of the molecular mechanisms underlying tumorigenesis is a necessary step for the implementation of precision cancer medicine. Given that cancer development is an evolutionary process, the detection of signals of positive selection in the somatic mutational pattern of genes has been exploited to identify drivers across tumor cohorts. Specifically, the non-random spatial accumulation, or clustering, of mutations along the protein sequence has been used to identify cancer drivers and provide clues about oncogenic mechanisms (Chang *et al.*, 2016; Tamborero *et al.*, 2013a, Tokheim *et al*., 2016). This signal is complementary to others (such as recurrence and functional impact) and thus, their combination can produce more comprehensive lists of driver genes (Porta-Pardo *et al.*, 2017; Rheinbay *et al.*, 2017; Tamborero *et al.*, 2013b).

Since the rate of mutation generation across the genome is highly variable (Alexandrov *et al.*, 2013; Lawrence *et al.*, 2013; Polak, *et al.*, 2015; Schuster-Böckler and Lehner 2012; Stamatoyannopoulos *et al.*, 2009), clustering-based methods face the challenge of constructing an accurate background model of the distribution of mutations to correctly assess the significance of observed clusters. Ideally, such a model would include all the genomic position-dependent covariates of the mutation rate. Alternatively, one can locally simulate the same number of mutations as observed in the region following the probabilities of k-nucleotide context-dependent substitutions and assess whether the distribution of mutations along the region follows the expectation (Mularoni *et al.*, 2016). This background model is not affected by large-scale covariates of the mutation rate (e.g. replication timing or chromatin state) and can thus be applied to any region of the genome of any species.

Here we introduce OncodriveCLUSTL, a new linear clustering algorithm to detect genomic regions and elements with significant clustering signals. The algorithm is based on a local background model derived from the observed tri- or penta-nucleotide substitution frequency of a cohort. OncodriveCLUSTL identifies known mutation clusters and driver genes across TCGA cohorts. It outperforms the existing OncodriveCLUST (Tamborero *et al.*, 2013a), and complements methods based on different signals of positive selection. We show that OncodriveCLUSTL identifies mutation clusters in human promoter regions and in mouse genes.

## 2 Implementation and availability

**Fig. 1.**
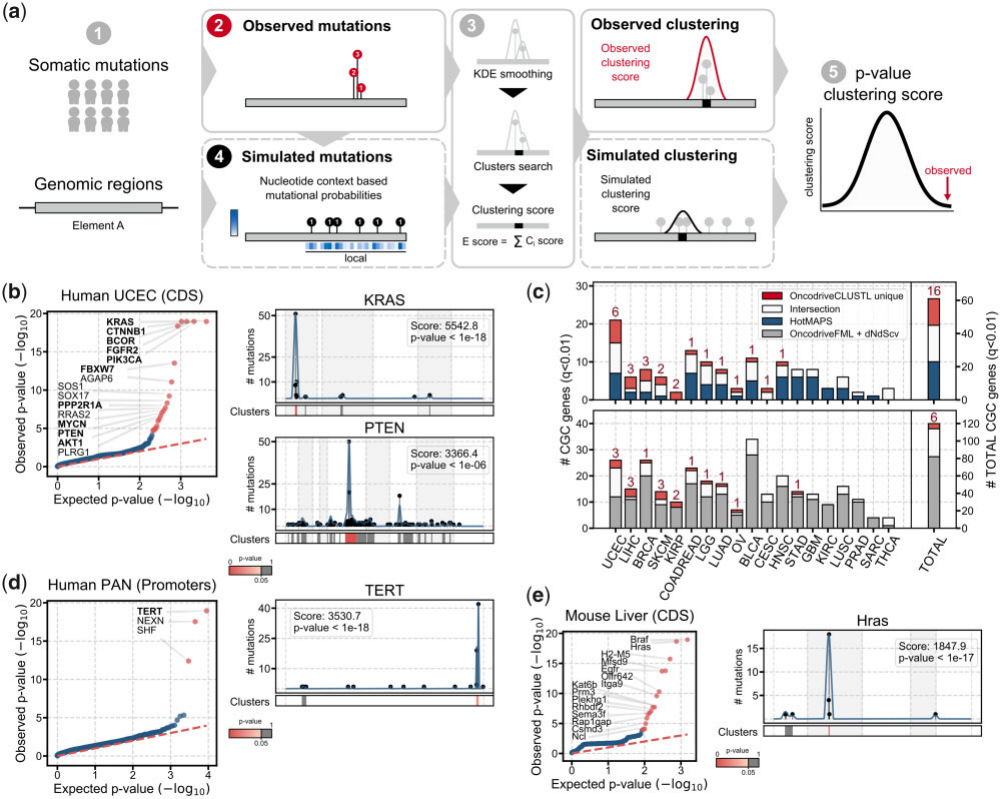
OncodriveCLUSTL algorithm and results. Overview of OncodriveCLUSTL (**a**). OncodriveCLUSTL detects well-known cancer genes (**b**) and complements methods based on different signals of positive selection (**c**). OncodriveCLUSTL can be successfully applied to mutations in promoter regions (**d**) and mouse genes (**e**)

OncodriveCLUSTL is an unsupervised clustering algorithm implemented in Python 3.5. It analyzes somatic mutations that have been observed in genomic elements (GEs) across a cohort of tumor samples (Fig. 1a-1). Mutations in each GE are smoothed along its sequence using a Tukey-based kernel density function, and clusters are identified (Fig. 1a-2, 3) and scored based on the number and the shape of the distribution of mutations. Cluster scores are summed up to produce a GE clustering score. The significance of the observed clusters and GEs is assessed through the analysis of *n* iterations, where mutations are randomly sampled within a window of nucleotides centered at each mutation (local), following the frequency of cohort tri- or penta-nucleotide changes (Fig. 1a-4, 5; Supplementary Methods for further details). By default, *P*-values are adjusted using the Benjamini-Hochberg method and GEs below 1% false-discovery rate (FDR) are considered significant. OncodriveCLUSTL source code and examples are freely available at https://bitbucket.org/bbglab/oncodriveclustl. A web version of OncodriveCLUSTL can be run at https://bbglab.irbbarcelona.org/oncodriveclustl.

## 3 Performance

### 3.1 Mutations in human protein-coding genes across 19 TCGA cohorts

OncodriveCLUSTL detects well-known cancer genes in the COSMIC Cancer Gene Census (CGC; Sondka *et al.*, 2018) with clusters of different sizes (Fig. 1b;Supplementary Figs S3 and S8; Supplementary Table S2 and S3) (Ellrott *et al.*, 2018). It outperforms the previously developed protein-clustering method OncodriveCLUST (Tamborero *et al.*, 2013a), which builds a background model obtained from synonymous mutations, in both true and false positives rates (Supplementary Figs S4, S8 and S9; Supplementary Methods for further details). These findings demonstrate that the improved clustering detection method and the local background model fine-tune the detection of drivers. OncodriveCLUSTL also exhibits similar specificity and sensitivity as the 3D protein-clustering method HotMAPS (Tokheim *et al.*, 2016) (Fig. 1c, Supplementary Figs S5 and S8–S11). Interestingly, although the linear clustering analysis performed by OncodriveCLUSTL can miss the detection of 3D clusters (Supplementary Fig. S10), it can identify CGCs with clusters of truncating or silent mutations (Supplementary Fig. S10) as well as CGCs without a PDB structure or protein model (Supplementary Fig. S11), which are missed by HotMAPS. In addition, the results of OncodriveCLUSTL complement those of methods based on distinct signals of positive selection (OncodriveFML, Mularoni *et al.*, 2016; dNdScv, Martincorena *et al.*, 2017) (Fig. 1c, Supplementary Figs S6 and S7), thus highlighting the relevance of combining methods exploiting different signals to enhance comprehensiveness in driver’s identification.

### 3.2 Mutations in promoters across a cohort of tumor whole-genomes

Consistent with the study describing the dataset (Fredriksson *et al.*, 2014), OncodriveCLUSTL found a significant cluster in the TERT promoter (Fig. 1d), the mutations of which result in the upregulation of TERT (Supplementary Fig. S12). Significant clustering was also detected in few other promoters, which need to be carefully vetted to be nominated as cancer drivers, as we and others have shown that some local mutational processes can also lead to mutation clustering (Sabarinathan *et al.*, 2016; Zou *et al.*, 2017).

### 3.3 Mutations in C3H mouse genes in chemically induced hepatocarcinomas

As described by the authors of the dataset (Connor *et al.*, 2018), OncodriveCLUSTL identified significant clustering in Braf, Hras and Egfr (Fig. 1e).

## 4 Conclusions

OncodriveCLUSTL is a new method to identify sequence-based clustering signals across the genome. It shows satisfactory sensitivity and specificity, outperforming the existing OncodriveCLUST and complementing other methods of driver detection in coding sequences. It can also be successfully applied to the detection of mutational clustering in non-coding regions and in non-human data.

## Supplementary Material

btz501_Supplementary_Data
