# OncodriveCLUSTL: a sequence-based clustering method to identify cancer drivers

**DOI:** 10.1093/bioinformatics/btz588

**Published:** 2019-08-01

**Authors:** Claudia Arnedo-Pac, Loris Mularoni, Ferran Muiños, Abel Gonzalez-Perez, Nuria Lopez-Bigas

*Bioinformatics* (2019) doi: 10.1093/bioinformatics/btz501

In the above article the final acknowledgement in the funding statement on page 3 has been changed to read ‘C.A.-P. is supported by “la Caixa” Foundation (ID 100010434) with code [LCF/BQ/ES18/11670011]’

The author apologises for this error.

