# Supplementary material for: OncodriveCLUSTL: a sequence-based clustering method to identify cancer drivers
Source: Bioinformatics. 2019 Jun 22;35(22):4788–90. doi: 10.1093/bioinformatics/btz501 (PMC6853674; doi:10.1093/bioinformatics/btz501)
Supplement: btz501_Supplementary_Data [file btz501_supplementary_data.zip › btz501-suppl_data/supplementary_material.pdf]

Supplementary Material

OncodriveCLUSTL: a sequence-based clustering method to identify cancer drivers

Claudia Arnedo-Pac[1], Loris Mularoni[1], Ferran Muiños[1], Abel Gonzalez-Perez[1,2] and Nuria Lopez-Bigas[1,2,3#]

[1]Institute for Research in Biomedicine (IRB Barcelona), The Barcelona Institute of Science and Technology (BIST), Barcelona, Spain.
[2]Research Program on Biomedical Informatics, Universitat Pompeu Fabra, Barcelona, Spain.
[3]Institució Catalana de Recerca i Estudis Avançats (ICREA), Passeig Lluís Companys 23, 08010 Barcelona, Spain
[#]To whom correspondence should be addressed.

Index

1. Supplementary Methods......3
- Tumor mutation datasets......3
- Coordinates of genomic elements......3
- Implementation......4
- Cancer Gene Census list......4
- List of false positives genes......4
- OncodriveCLUSTL methodological details......5
- OncodriveCLUSTL model selection......10
- Running examples......12
- Benchmark......14
- Cluster plots......15
- Expression analysis......15
- Code availability......16

2. Supplementary Figures......17

3. Supplementary Tables......29

4. Supplementary References......30

1 Supplementary methods

Tumor mutation datasets

We obtained the mutations identified in primary tumors from three different datasets: 19 whole exome sequencing (WXS) cohorts of The Cancer Genome Atlas (TCGA) project (Ellrott *et al.*, 2018) (downloaded on December 29th, 2016), 14 whole genome sequencing cohorts from TCGA WGS-505 project (Fredriksson *et al.*, 2014) (downloaded on March 31st, 2015), and a WXS dataset (European Nucleotide Archive ERZ537501) of chemically-induced C3H mouse liver tumors (Connor *et al.*, 2018) (downloaded on October 11th, 2018). TCGA WXS cohorts consist of 8,263 samples (1,382,259 substitutions) from 19 different cancer types, each of them composed by a minimum of 200 samples. Hypermutated tumors were removed from the datasets. To define hypermutated samples for a given cohort, we calculated the distribution of the number of alterations across samples in the cohort. Those samples bearing a minimum number of 1,000 alterations exceeding 1.5 times the interquartile range above the 75th percentile were considered hypermutated. TCGA WGS-505 consist of 505 samples (12,423,016 substitutions) from 14 different cancer types. A whole-genome Pancancer cohort was obtained by merging all cohorts in the TCGA WGS-505 dataset. Original mouse WXS dataset consist of substitutions identified across 78 liver tumors of C3H mice generated spontaneously or by exposure to diethylnitrosamine (DEN). For our analysis, we selected DEN-exposed tumors (32,494 substitutions).

Coordinates of genomic elements

The hg19 genomic coordinates of human protein coding genes (n=20,098) were obtained from ENCODE (http://www.gencodegenes.org) using GENCODE release v.19 (ftp://ftp.ebi.ac.uk/pub/databases/gencode/Gencode_human/release_19/gencode.v19.annotation.gtf.gz). Only coding sequences (CDS) of protein coding transcripts (‘gene_type’ and ‘transcript_type’ annotated as ‘protein-coding’) were retrieved as previously described in Mularoni *et al*., 2016. Promoter regions (n = 20,096) were obtained by mapping the 2500 bp sequences immediately upstream the transcription start sites (TSS) of protein-coding genes. All nucleotides that corresponded to protein-coding sequences, untranslated regions (UTRs) or short intronic splice sites were removed (Mularoni *et al.*, 2016). C3H mouse genomic coordinates for protein coding genes (n=19,234) version C3H_HeJ_v1.86 were downloaded from Ensembl release 90 (ftp://ftp.ensembl.org/pub/release-90/gtf/mus_musculus_c3hhej/Mus_musculus_c3hhej.C3H_HeJ_v1.86.chr.gtf.gz). Only CDS of protein coding transcripts (‘feature’ and ‘gene_biotype’ annotated as ‘CDS’ and ‘protein_coding’, respectively) were retrieved. Overlapping elements of the same type (CDS or promoters of the same gene) were merged together. Genomic elements showing incorrect annotations were manually removed from the datasets.

Implementation

OncodriveCLUSTL is implemented as a Python 3.5 package. It depends on external Python libraries including *bgparsers, bgreference*, *bgsignature*, *click*, *daiquiri*, *intervaltree*, *matplotlib*, *numpy*, *pandas*, *scipy*, *statsmodels* and *tqdm*. OncodriveCLUSTL takes as input: i) a TSV file containing mutational data from multiple samples of either WXS or WGS and ii) a TSV annotations file containing the genomic positions of the genes or other genomic elements to be analyzed. OncodriveCLUSTL generates two main output files: i) 'elements_results.txt' contains a list of genomic elements ranked by the significance of their clustering signal; ii) 'clusters_results.tsv' lists the information of all clusters found in genomic elements deemed significant. For a detailed explanation on how to run OncodriveCLUSTL please check the README document at https://bitbucket.org/bbglab/oncodriveclustl.

Cancer Gene Census list

We obtained the latest version of the COSMIC Cancer Gene Census (CGC) list containing a total of 719 genes (https://cancer.sanger.ac.uk/census) (downloaded on October 15th, 2018) (Sondka *et al.*, 2018). Among them, 574 and 145 genes were classified in COSMIC Tier 1 and Tier 2, respectively. Tier 1 genes are those genes with solid available evidence of activity relevant to cancer as well as evidence of mutations in cancer that promote oncogenic transformation; Tier 2 genes correspond to those with strong indications of a role in cancer but with less extensive available evidence.

List of false positives genes

We generated a list of potential false positive genes (or "fishy" genes) that for reasons other than their involvement in tumorigenesis tend to be frequently mutated in tumors (Przytycki and Singh, 2017; Lawrence *et al.*, 2013). The list includes very long genes, olfactory receptors and non-expressed genes for each TCGA cohort. The list of long genes (n=6) included TTN, OBSCN, GPR98, HMCN1, RYR2, and RYR3. Olfactory receptors (n=857) were obtained from The Human Olfactory Data Explorer (Safran *et al.*, 2003) at https://genome.weizmann.ac.il/horde/ (downloaded on February 14th, 2018). Expression data was obtained from TCGA (https://gdc.cancer.gov/about-data/publications/pancanatlas) (downloaded on April 7th, 2016). Non-expressed genes were defined as genes with RNA-seq expression estimation smaller or equal to 0 (log2 scale) in 80% or more samples in the cohort.

OncodriveCLUSTL methodological details

OncodriveCLUSTL analyzes clustering signals in nucleotide sequences from human and non-human data. It has been tested using the hg19 and GRCh38 reference human genomes, c3h, mm10, cast and car mouse genomes, and f344 rat genome obtained via the *bgreference* package (https://bitbucket.org/bgframework/bgreference). In addition, OncodriveCLUSTL can virtually be run using data from any reference genome provided that it is compiled through the *bgreference* package.

Input data parsing. By default, OncodriveCLUSTL only requires two main inputs: i) mutations file, a TSV file containing substitutions identified across a cancer whole exome (WXS) or whole genome (WGS) sequencing cohort, assuming all SNVs are mapped to the positive strand; ii) annotations file: a TSV file with the genomic positions annotations of the genes or other genomic elements (GEs) to be analyzed (details in README). Of note, genomic coordinates for one element cannot overlap. By default, OncodriveCLUSTL analyzes all GEs provided in the annotations file. Alternatively, OncodriveCLUSTL can limit the analysis to a number of user-specified GEs (details in README). To start the analysis, OncodriveCLUSTL first parses the GEs coordinates from the annotations file. GE mutations are obtained by intersecting its genomic coordinates with those of substitutions (SNVs). Therefore, only those mutations falling inside the GE coordinates will be taken into account for clustering signals calculation.

Nucleotide context mutational probabilities calculation. The significance of clustering signals detected by OncodriveCLUSTL relies on a background model calculated from the input cohort k-mer nucleotide SNVs. OncodriveCLUSTL can compute tri-nucleotide or penta-nucleotide mutational profiles in two different modalities (option ‘signature-calculation’):

1) ***Relative mutation frequencies***. By default, OncodriveCLUSTL calculates the mutational profile as the relative mutation frequencies of each k-mer nucleotide SNVs with respect to the total number of SNVs in the input cohort. Briefly, a dictionary is constructed including all possible reference k-mers and 3 possible alternates of each of them (192 for tri-nucleotides, 3072 for penta-nucleotides). Following the methodology laid out in Mularoni *et al*. (2016), mutational probabilities of each reference k-mer to alternate pair are computed as the number of SNVs observed for the reference-alternate pair out of the total number of SNVs analyzed in the cohort or cancer type (Mularoni *et al*., 2016). To this end, OncodriveCLUSTL takes into account only those input SNVs whose reference k-mer sequence does not contain a sequence gap or unannotated nucleotides in the reference genome.

2) ***Normalized mutation frequencies***. Alternatively to relative mutation frequencies, OncodriveCLUSTL can normalize k-mer nucleotide SNVs counts by the k-mer nucleotide reference counts in the regions provided by the user. Briefly, mutational probabilities of each reference k-mer to alternate pair are computed as the number of SNVs observed for the

reference-alternate pair out of the total number of reference k-mer nucleotides found in the regions listed in the annotations file. In this case, only mutations that fall inside the regions will contribute to the signature calculation. Input SNVs whose reference k-mer sequence contains a sequence gap or unannotated nucleotides in the reference genome are skipped. This calculation can be of interest when analyzing protein-coding genes from a WXS cohort, where the annotation file contains all protein-coding genes annotations. Of note, users must ensure that the annotations file contains enough genomic elements for an accurate mutational profile calculation.

By default, OncodriveCLUSTL calculates either of the previously specified mutational profiles under the assumption that all samples come from the same group (e.g., cancer type). Alternatively, OncodriveCLUSTL can calculate one specific mutational profile for each group contained in the mutations file (details in README) and randomize each mutation according to it. This option can be applied to split mutational profiles by different cancer types in a Pancancer mutation file or by delimited mutational processes affecting a cancer cohort. Of note, it is important that users warrant that the number of mutations for each group is sufficient for an accurate mutational profile calculation.

In addition, OncodriveCLUSTL is prepared to run using a mutational profile provided by the user (details in README). This option can be of interest to calculate more complex mutational profiles or when the number of mutations in the input cohort is not large enough.

In our analysis, we pre-calculated mutational profiles for OncodriveCLUSTL as follows: i) for human coding regions, tri- or penta-nucleotide (Supp. Table 1) SNVs counts in the cohort were normalized by the corresponding k-mer nucleotide counts in the hg19 protein-coding regions file. SNVs in cancer genes (COSMIC CGC list), falling outside coding regions, or whose reference k-mer sequence contained a sequence gap or unannotated nucleotide were skipped; ii) for human promoters, tri-nucleotide SNVs counts for each of the cancer types in the Pancancer file were normalized by tri-nucleotide counts in the hg19 whole genome. Therefore, a mutational profile was obtained for each of the cancer types in the Pancancer file. SNVs in cancer genes (COSMIC CGC list) or whose reference k-mer sequence contained a sequence gap or unannotated nucleotide were skipped; iii) for mouse coding regions, tri-nucleotide SNVs counts in the cohort were normalized by the tri-nucleotide counts in the c3h protein-coding regions. SNVs falling outside coding regions, or whose reference k-mer sequence contained a sequence gap or unannotated nucleotides were skipped.

Clustering analysis. The following analysis was restricted to those GEs in the annotations file that contained 2 or more SNVs mapping the mutations file. We proceeded in the following steps:

1) ***Analysis of observed mutations***. OncodriveCLUSTL conducts linear clustering analysis of the mutations observed along the GE sequence provided by the user. Two alternative

modalities are available: i) by concatenating the connected components of the GE supplied (e.g., glueing the consecutive ends of the CDS chunks in the exons of a given gene) in which case we increase the method's sensitivity towards clusters in expanding the boundaries of the collapsed regions or ii) analyzing the connected components of the GE separately (by default). For both modes, the following subsequent steps are carried out:

(1a) ***Smoothing***. We used a Tukey kernel to smooth the function *n* that maps each position of the GE to the number of mutations observed at that position. Of note, the smoothing is carried out in the position coordinates implied by the chosen modality (concatenated GE components or not). Bearing that in mind, the smoothing function *S* is defined for each position *p* as:

$$S(p)=\frac{1}{M}\sum_{i=-L}^{L} n(p+i)T(\frac{i}{L});$$

where *T* is the Tukey function defined as:

$$T(x)=(max\{1-x^2,0\})^2;$$

M is the total mass spread by the kernel:

$$M=\sum_{i=-L}^{L} T(\frac{i}{L})\sim L\int_{-1}^{1} T(x)dx;$$

and *L* is the half window length where the smoothing is applied. The smoothing window length is 11 bp long by default.

(1b) *Root clusters generation*. Upon smoothing, root clusters of the GE are computed using the function *S,* which is defined along the GE's sequence. A cluster is always defined as 3 positions x-y-z in the linear sequence. For each local maxima m of S in the linear sequence we will define its root cluster as follows: i) if m is neither the first nor last position of the GE, then y=m, and x and z are as the closest local minima surrounding a local maxima y of *S;* ii) if m is the first positon of the GE, then x=y=m and z is the first local minima; iii) if m is the last position of the GE, then y=z=m and x is the last local minima.

(1c) *Merging of clusters*. In this step, the algorithm recursively identifies clusters that are closer than a given gap length (clustering window) and merges them in one new unified cluster. Starting from the 5'-most cluster in the GE defined as x-y-z, the algorithm looks for 3' adjacent clusters x'-y'-z'. The clusters merge if the distance between z and y' is

smaller than or equal to the clustering window length. Then, a new cluster is obtained by merging both root clusters. The 5' and 3' boundaries of the new cluster are x and z', respectively. The maximum of the new cluster corresponds to the maximum with highest smoothing score amongst the root clusters, y or y'. When y and y' have equal scores, if they are contiguous positions y is selected as the new clusters maximum; otherwise, the clusters are not merged. The search iterates until no further merging is possible. The clustering window length is 11 bp long by default.

(1d) *Scoring of clusters*. A score is assigned to each cluster based on the number of SNVs it contains and their distribution across the cluster as follows:

$$Score = N \cdot \sum_i f_i \cdot 2^{-d_i/2};$$

where *N* is the total number of SNVs mapping to the GE; *i* runs through all mutated positions in the cluster; $f_i = 100 \cdot (m_i / N)$ is the percentage of mutations observed at position *i* with respect to *N;* and $d_i = |i - i_{max}|$ is the distance from *i* to the position reaching the maximum value of the smoothing function in the cluster. Given two clusters with the same number of mutations, this formula favours the one with the mutations concentrated in fewer positions. Clusters with fewer mutations than the defined threshold (2 by default) are scored to 0.

(1e) *Scoring of the GE*. The clustering score of a GE corresponds to the sum of the scores of its clusters.

2) ***Analysis of simulated mutations***. The same number of mutations observed in an GE are randomly sampled several times with replacement. The probability that a mutation is placed in a given position is derived from the mutational profile of the cohort, which is computed as explained in the section *Nucleotide context mutational probabilities calculation*. Independently of the modality of observed mutations analysis (concatenated GE regions or not), mutations are randomized in the reference genomic sequence as follows. At the time of distribution of each mutation, a window of nucleotides (simulation window) centered at the mutated nucleotide is defined by default. The simulated mutation is therefore distributed at any position within the simulation window, which is 31 bp by default but can be extended up to 101 bp. When a mutation is observed close to the region's boundaries, the simulation window centered at the mutated nucleotide can expand outside the region analyzed (e.g., if a mutation is found in the last position of an exon and the simulation window is 31 bp long, 15 bp of the window will fall within the adjoining intron). However, simulated mutations that fall outside the GE are discarded during the simulated clustering analysis and therefore do not contribute to the simulated clusters and GE's scores, which ultimately affects the significance of the observed clustering signals. As an alternative, if specified, OncodriveCLUSTL allows to displace the simulation window to fit

inside the analyzed region in those cases where a subset of bp of the simulated window fall outside the analyzed region (e.g., if a window extends part of an exon and an intron, it can be placed to fit inside the exon). Displaced simulation windows maintain the defined window length but do not respect the central position for the observed mutation. For those cases where the length of the simulation window is greater than the region analyzed, the simulation window is trimmed to the region start and end. Of note, in this mode, those simulation windows that do not expand regions boundaries keep the observed mutation in the central position. In our analysis, all simulations were done restricting simulation windows to the region studied, since expanding simulation windows may not account for different yet known or unknown mutational processes acting on regions of different nature (e.g., exons and introns, Frigola et al., 2017). This strategy may reduce the number of such clustering artifacts, although the significance of the observed clustering signals might be underestimated. This can be the case for small GE regions. As OncodriveCLUSTL allows both simulation strategies, we encourage users to test which is the most suitable according to their data by analyzing the distribution of the significance of the clustering signals. To this aim, OncodriveCLUSTL can export quantile-quantile plots of the generated results. Once all mutations are randomly distributed across their respective simulation windows, OncodriveCLUSTL infers the clustering (steps 1a-1e) for the randomly generated mutations using the same analysis mode as in observed mutations (concatenated GE or not). Each iteration of random distribution of mutations (1,000 in all analyses described) retrieves simulated clusters and GE's scores.

P-value computation and multiple test correction. OncodriveCLUSTL generates three p-values per GE. First, an empirical p-value is computed as the fraction of iterations that yield a simulated GE score greater to or equal than the observed GE score. Second, an analytical p-value is calculated by fitting simulated GE scores to a gaussian kernel density estimate distribution and deriving the upper quantile of the observed GE score. Third, a second analytical p-value corresponding to the top-scoring cluster of the GE is computed following the same approach, fitting the distribution of the simulated cluster scores. To reduce the burden of analytical p-values computations, the algorithm randomly samples a subset of 1,000 simulated GEs over the total simulated GEs scores if the number of simulations is greater than 1,000; likewise, 1,000 simulated cluster scores are randomly sampled when the number of simulated clusters exceeds 1,000. All resulting p-values are subsequently adjusted (q-values) using the Benjamini-Hochberg method at 1% false-discovery rate (FDR). In other words, GEs with q-value < 0.01 are considered potential drivers. All results shown here are based on rankings of GE scores analytical p-values.

OncodriveCLUSTL model selection

Window hyperparameters: smoothing, clustering and sampling. OncodriveCLUSTL is an unsupervised method to identify clustering of mutations along the genomic sequence of GEs. The method should raise a clustering signal whenever the clustering observed departs from what it would be expected assuming that the mutations are generated under neutral evolution. However, the method resorts to three main hyperparameters that strongly determine its performace: the shape of identified clusters depends on the smoothing (i) and clustering (ii) windows; the simulation of mutations depends on a sampling or simulation window (iii) which defines the region where mutations are randomly distributed. On the one hand, large smoothing and clustering windows tend to generate large clusters for either observed and simulated mutations; on the other hand, large simulation windows tend to spread simulated mutations along the sequence, which decreases the likelihood of formation of simulated clusters. The interplay between smoothing, clustering and sampling windows determines what kind of clustering signals the unsupervised method is bound to identify. Changes to the distribution of GEs p-values may end up affecting their goodness of fit to the uniform distribution.

We devised a strategy for model selection based on two criteria: i) goodness of fit of observed p-values vs. the uniform distribution; ii) enrichment of *bona-fide* known cancer elements in the ranking given by the method as an output. For each dataset, we ran OncodriveCLUSTL over all possible combinations in a predefined grid of hyperparameter values and selected the best configurations according to these criteria.

Goodness-of-fit. For each configuration, we want to test whether the distribution of observed p-values is similar to the theoretical distribution of p-values, i.e., the uniform distribution in the interval [0, 1]. To this end, we computed the Kolmogorov-Smirnov (KS) goodness-of-fit statistic with respect to the uniform distribution in the interval [0, 1]. The KS statistic is defined in terms of two cumulative probability functions: i) the one arising from the observed data (empirical cumulative probability, ECP):

$$ECP(x)=\frac{1}{n}\sum_{i=1}^{n} 1_{(-\alpha,x]}(x_i);$$

And ii) the one arising from the uniform distribution (theoretical cumulative probability, TCP), which is linear. In order to proceed: first, we take the subset of observed p-values which are greater than 0.1; second, we randomly sampled 1,000 of them to avoid sample size biases in our comparisons; third, we calculated the KS statistic, which essentially measures the size of the maximum gap (deviance) between the observed and theoretical cumulative probability functions. To distinguish between p-value deflation and inflation, we computed whether the number of p-values above a threshold $\alpha=0.1$ was greater (inflation) or lower (deflation) than expected. Thus we defined a signed version of the KS statistic which is positive for the inflated and negative for the deflated. We selected all configurations bearing an absolute value of the

KS statistic up to 10% larger than the minimum KS statistic. This procedure left us with a set of most suitable configurations.

Enrichment in bona-fide known cancer elements. For each configuration we calculated a CGC genes enrichment score for the top ranking genes (n=40). For each $1 \leq n \leq 40$, we computed the proportion of CGC genes (C for short) within the subset of genes with rank *n* or lower, hereinafter S_n. Then, we added up all these proportions, albeit giving more weight to the terms arising from smaller sets. Hence, we computed the following enrichment score:

$$E = \sum_{n=1}^{40} \frac{1}{\log_2(n+1)} \cdot \frac{|S_n \cap C|}{|S_n|}$$

The configuration with highest enrichment score (E) was selected. For cohorts where this model selection strategy could not provide accurate models, we explored addtional values combinations of the before mentioned hyperparameters and manually curated the model selection on case-by-case basis using the approach described above. We hypothesize that the differences in performance could result from differential mechanisms shaping the mutational landscape for each cancer type (Alexandrov *et al.*, 2013). Therefore, we advice users to carry out model selection according to their data specificities and constraints. Information of all the adjusted hyperparameters for all analyzed TCGA cohorts can be found in Supp. Table 1. The functions used to generate these data are available at https://bitbucket.org/bbglab/oncodriveclustl. For those cohorts where the CGC enrichment could not be applied (e.g., promoter regions, mouse data), model selection was based on the goodness-of-fit (Supp. Table 1).

We illustrate the performance of the KS test with the following example. We calculated the empirical cumulative distribution of p-values obtained by different configurations of the TCGA WXS UCEC cohort, using smothing windows of 11, 15, 21, 25, 31, 35, 41 and 45 bp; clustering windows of 11, 15, 21, 25 and 31 bp; and simulation windows of 31 and 35 bp. We randomly sampled n=100 p-values and calculated the ECPs between 0.1 and 1 for each of the configurations (Supp. Fig. 1). Finally, the best configuration was choosen as the one with higher CGC enrichment, as explained.

Background mutational probabilities. To test which of the k-mer context mutational probabilities, tri-nucleotides or penta-nucleotides, was able to generate more accurate models, we ran OncodriveCLUSTL using tri- and penta-nucleotde contexts with the 19 selected TCGA WXS datasets, keeping the rest of parameters as default. We calculated the Kolmogorov-Smirnov (KS) statistic to assess the fitness of the observed distribution of p-values to the expected uniform distribution (Supp. Fig. 2). For these data, we found no clear differences between the KS statistic obtained from tri- or penta-nucleotide based background models. Given that tri-nucleotide mutational probabilities are less computationally expensive, we set them as

the default. However, recently Martincorena and colleagues have shown that penta-nucleotide contexts can explain more accurately the mutational processes in melanomas caused by UV light (Martincorena *et al.*, 2017). Consequently, all analyses shown in the main paper were carried out using tri-nucleotide mutational probabilities, except for the melanomas cohort, to which the penta-nucleotide mutational probabilities was computed. We recommend users to conscientiously select OncodriveCLUSTL tri- or penta-nucleotide probabilities that best fit their own mutational datasets.

Quantile-quantile plots. In order to evaluate the models generated by OncodriveCLUSTL, we generated quantile-quantile plots (QQ-plots) comparing the observed p-value distribution (y-axis) with the expected uniform p-value distribution (x-axis). We consider a model to be accurate if the observed p-values closely fit the uniform distribution (red dash line) for the most part of the GEs analyzed, i.e., those GEs not bearing a significant clustering signal (red dots, $q < 0.01$). The names of top-ranking genes are shown. The names of gene symbols annotated in the CGC appear in bold. The code used to generate QQ-plots is available at https://bitbucket.org/bbglab/oncodriveclustl and can be automatically run together with the clustering analysis of a cancercohort using the command line (details in README).

Running examples

Complete guideliness on how to run OncodriveCLUSTL can be found in the README doument at https://bitbucket.org/bbglab/oncodriveclustl. Briefly, we highlight here different running options of the algorithm that can be selected through the command line using OncodriveCLUSTL version 1.1.0 as described in the manuscript:

Default run

~$ oncodriveclustl -i /INPUT_PATH/mutations_file.tsv -o /OUTPUT_PATH/output_directory -r /INPUT_PATH/regions_file.tsv.gz

By default, OncodriveCLUSTL analyzes all genomic elements in ‘regions_file.tsv.gz’. It assumes that genomic coordinates and mutations are mapped to hg19 human reference genome. The clustering analysis is performed over all elements with at least 2 SNVs; clusters are defined by a minimum number of 2 SNVs. For each element, coordinates are analyzed separately, therefore no clusters are found expanding two regions. The smoothing and clustering windows are 11bp. One tri-nucleotide mutation frequencies are calculated for the whole input cohort in ‘mutations_file.tsv’. Simulation windows are 31bp; by default windows are centered on the observed mutation. Therefore mutations can be simulated a maximum of 15bp away from the margins of the region under analysis. Given that the default parameters may not be satisfactory for the cohort under analysis, a warning message is raised to underline that OncodriveCLUSTL

is running in default mode.

Human coding sequences using default parameters

~$ oncodriveclustl -i /INPUT_PATH/mutations_file.tsv -o /OUTPUT_PATH/output_directory -r /INPUT_PATH/regions_file.tsv.gz --concatenate

The ‘concatenate’ flag links together genomic regions of an element. As a consequence, clusters can be found expanding two genomic regions. Of note, this option was developed for the analysis of protein coding genes, where different regions correspond to different coding sequences in a gene.

C3H mouse coding sequences using default parameters

~$ oncodriveclustl -i /INPUT_PATH/mutations_file.tsv -o /OUTPUT_PATH/output_directory -r /INPUT_PATH/regions_file.tsv.gz --concatenate --genome c3h

The ‘genome’ command allows to change the reference genome of the data under analysis.

Human coding sequences using non-default parameters

~$ oncodriveclustl -i /INPUT_PATH/.mutations_file.tsv -o /OUTPUT_PATH/output_directory -r /INPUT_PATH/regions_file.tsv.gz --concatenate --smooth-window 35 --cluster-window 15 --kmer 5 --simulation-window 35 --simulation-mode region_restricted --element-mutations 3 --cluster-mutations 3 --signature-calculation region_normalized

Smoothing, clustering and simulation windows can be set using the ‘smooth-window’, ‘cluster-window’ and ‘simulation-window’ commands. The k-mer nucleotide context to calculate the mutational profile can be changed to penta-nucleotides using ‘kmer’ command. Simulated mutations can be forced to fall inside the region under analysis through the ‘simulation-mode’ command. Elements and clusters mutation thresholds are set to 3 SNVs. The mutational profile is calculated as penta-nucleotide SNVs counts normalized by penta-nucleotide reference counts in the input regions file ‘regions_file.tsv.gz’.

Pancancer human coding sequences using default parameters

~$ oncodriveclustl -i /INPUT_PATH/mutations_file.tsv -o /OUTPUT_PATH/output_directory -r /INPUT_PATH/regions_file.tsv.gz --concatenate --signature-group CANCER_TYPE

A mutational profile will be calculated for each group included in the ‘CANCER_TYPE’ column provided in the ‘mutations_file.tsv’.

Benchmark

The performance of OncodriveCLUSTL in coding regions was first assessed by computing the enrichment of CGC genes (Supp. Methods) identified by the method in the cohort under analysis. The enrichment was compared with two clustering methods: OncodriveCLUST (Tamborero *et al.*, 2013) and HotMAPS (Tokheim *et al.*, 2016). OncodriveCLUST method identifies significant clustering signals in the protein sequence using a background model based on the distribution of non-synonymous mutations, and can therefore be only applied to protein-coding genes. With respect to OncodriveCLUST, the main differences of our new algorithm include: i) the refinement of the background model calculation, now based on the k-mer nucleotide SNVs frequencies, which replaces the null model of OncodriveCLUST; ii) the construction of a local background model at the nucleotide level allows to extend the analysis to non-coding regions of the genome. On the other hand, HotMAPS is a 3D protein-clustering algorithm that analyzes clusters of missense mutations using a background model based on a discrete uniform distribution of mutations in the protein structure. The main differences with OncodriveCLUSTL are: i) as it uses 3D spatial information, HotMAPS can only analyze genes with experimentally solved protein structures or protein models, and therefore it cannot be applied to genes outside this set nor non-coding regions; ii) while HotMAPS only takes missense into account, OncodriveCLUSTL clustering is built upon all substitution mutations (missense, nonsense or silent). To compare their performances, we calculated the fold increase in the proportion of CGC genes among sets with increasing number of top ranking genes detected by each method. Briefly, for each set of increasing number of top ranking genes, we calculated the fraction between the proportion of GCG genes within the set and the proportion of CGC genes within all genes detected by the method. This correction accounts for the fact that methods differ in the number of genes analyzed, and therefore the proportion of GCG genes among them, which ultimately modifies the probabilities of detecting CGC genes. Enrichment plots show the enrichment of the top 40 ranking genes identified by OncodriveCLUSTL, OncodriveCLUSTL and HotMAPS. We next calculated the performance of these three methods in the detection of false positive genes (FP). To this aim, we generated a list of FP genes for each cohort (Supp. Methods) and calculated the fold enrichment in FP as explained, now correcting for the proportion in FP analyzed by each method. Finally, we generated FP enrichment plots for the top 40 ranking genes.

To show the complementarity between linear (1D) and non-linear (3D) clustering methods we calculated the number of CGC genes detected by OncodriveCLUSTL and HotMAPS ($q < 0.01$). For each cohort, we computed the number of unique CGC genes detected by OncodriveCLUSTL, HotMAPS, and the number CGC genes detected by both. To study in detail differences in CGC detection, we ran Variant Effect Predictor (VEP) tool (McLaren *et al.*, 2016) version 88 (hg19 reference genome) for input TCGA cohorts. For each mutation and alternate, we selected the highest consequence type estimated among all coding transcripts. We classified CGC genes detected according to their mode of action as described in The Catalog of

Cancer Genes (version 01/05/2017) at The Cancer Genome Interpreter (https://www.cancergenomeinterpreter.org/genes; downloaded on February 18th, 2019) (Tamborero *et al.*, 2018). In addition, we classified CGC genes according to their protein structure status: solved 3D structure (PDB identifier), protein model or none of them.

In parallel, we tested the complementarity of 1D linear clustering to other methods based on different signals of positive selection, including OncodriveFML (Mularoni *et al*., 2016) for functional impact bias and dNdScv (Martincorena *et al*., 2017) for recurrence. To this end, OncodriveCLUST version 1.0, HotMAPS version 1.1.3, OncodriveFML version 2.1.0 and dNdScv version 0.1.0 were run using default parameters.

Cluster plots

We generated the so called “cluster plots” to illustrate the distribution of mutations, smoothing curve and clusters along the sequence of a GE. The plot shows GEs sequence 5’-3’ (left to right) in the strand encoding them. For those GEs fragmented in different regions (e.g., exons in a gene), the sequence is shown as concatenated (x-axis). The different regions are delimitated by the different white-grey chunks and dashed lines. The first grid of the plot contains the number of mutations (left y-axis) mapped to their relative position in the concatenated GE. Smoothing curves (blue) correspond to the smoothed values per position following Tukey’s kernel density estimate application (right y-axis, labels not shown). GE score and p-value are highlighted in a box. The second grid illustrates observed clusters. Significant clusters are highlighted in a red-color scale where darker red corresponds to more significant p-values. Non-significant clusters are shown in grey. All functions needed to generate cluster plots are included in OncodriveCLUSTL code and can be run through the command line (details in README).

Expression analysis

Expression and copy-number data from TCGA WGS-505 dataset were obtained from Fredriksson *et al.* 2014. As described in the original article, RNA-sequencing (RNA-seq) BAM format data (hg19 assembly) and copy-number amplitudes (Affymetric SNP6 platform) of CDS (n=20,167) and lncRNAs (n=11,852) (GENCODE v17; Harrow et al., 2012) were processed following the methodology introduced by Akrami et al., 2013. Pancancer differential expression analysis between non-mutated and cluster-mutated samples was carried out for samples bearing a diploid copy number of the gene under analysis (log2 absolute amplitude < 0.2). Differences were assessed using U-Mann Whitney test ($\alpha=0.05$).

Code availability

OncodriveCLUSTL algorithm and a running example are available under GNU Affero General Public License at https://bitbucket.org/bbglab/oncodriveclustl. Results shown in the manuscript and supplementary material were calculated using OncodriveCLUSTL version 1.1.0 Further versions will be available through our repository. Additionally, a limited version of OncodriveCLUSTL can be run through our web at https://bbglab.irbbarcelona.org/oncodriveclustl.

2 Supplementary Figures

Supp. Fig. 1.

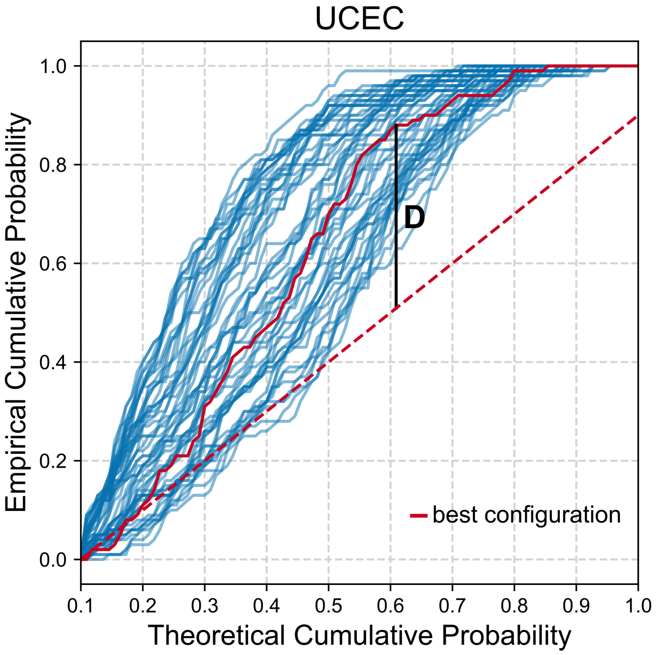

Supp. Fig. 1. Representation of the empirical cumulative probabilities of p-values for TCGA WXS UCEC cohort. For each configuration, the empirical cumulative probability of p-values was calculated and plotted against the theoretical uniform cumulative distribution between 0.1 and 1. The striped red line shows the expected uniform cumulative distribution. Results for the best configuration of parameters given the top 40 CGC enrichment are highlighted in red. The vertical black line shows the maximum deviance (D) of the best configuration. An alpha of 0.05 was used.

Supp. Fig. 2.

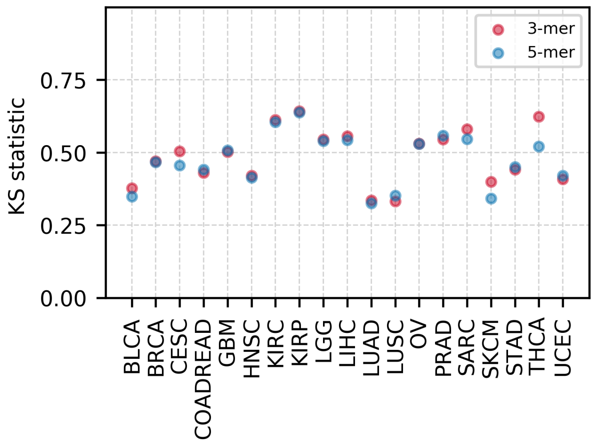

Supp. Fig. 2. Effect of tri-nucleotide or penta-nucleotide context mutational probabilities on OncodriveCLUSTL models. The Kolmogorov-Smirnov statistic showing the fitness of the observed p-values distribution to the expected uniform distribution was calculated for the 19 TCGA cohorts analyzed tri-nucleotide (3-mer, red) and penta-nucleotide (5-mer, blue) context relative mutational frequencies. Smoothing, clustering and simulation windows were ran as default.

Supp. Fig. 3.

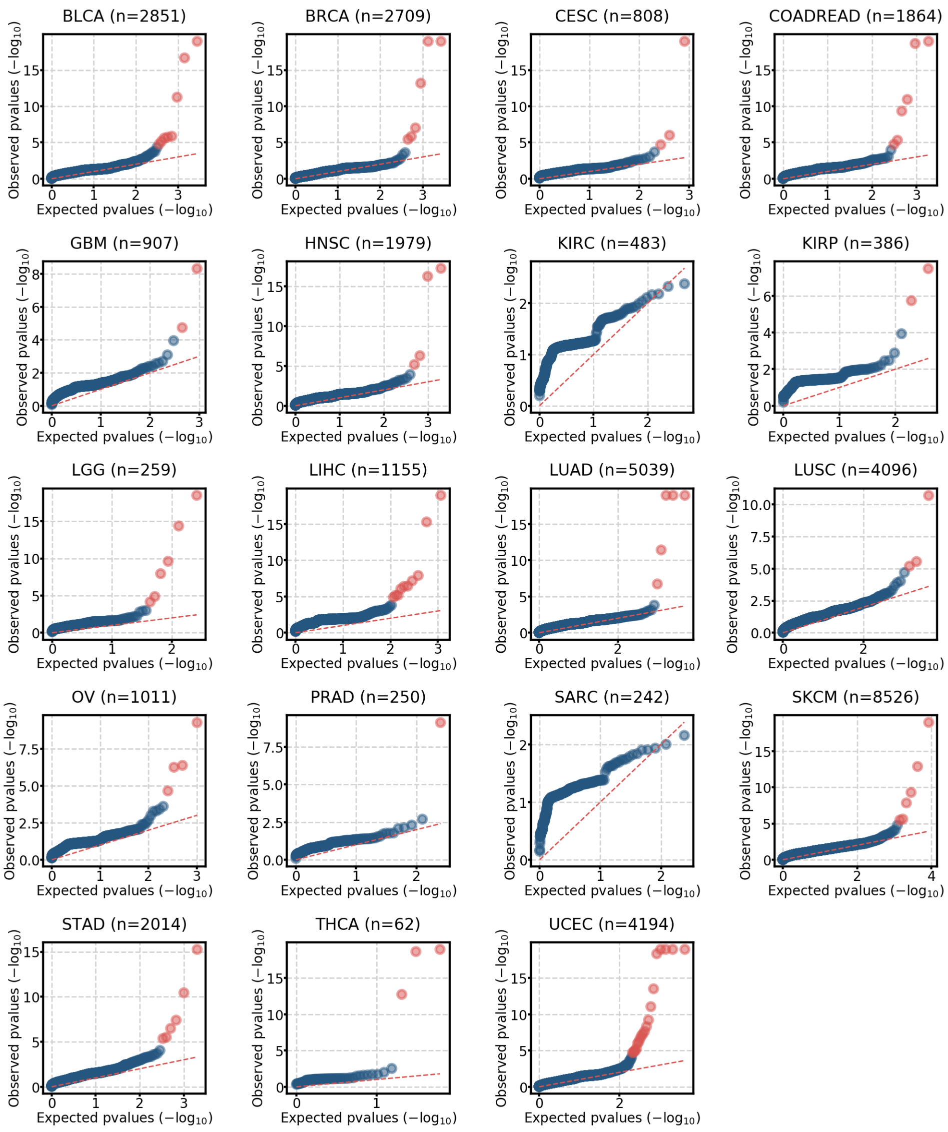

Supp. Fig. 3. Model adjustement by OncodriveCLUSTL. QQ-plots showing the observed p-values distribution versus the expected uniform distribution for all TCGA cohorts analyzed. Genes with q < 0.01 are highlighted in red. The number of p-values plotted on each QQ-plot is shown on top.

Supp. Fig. 4.

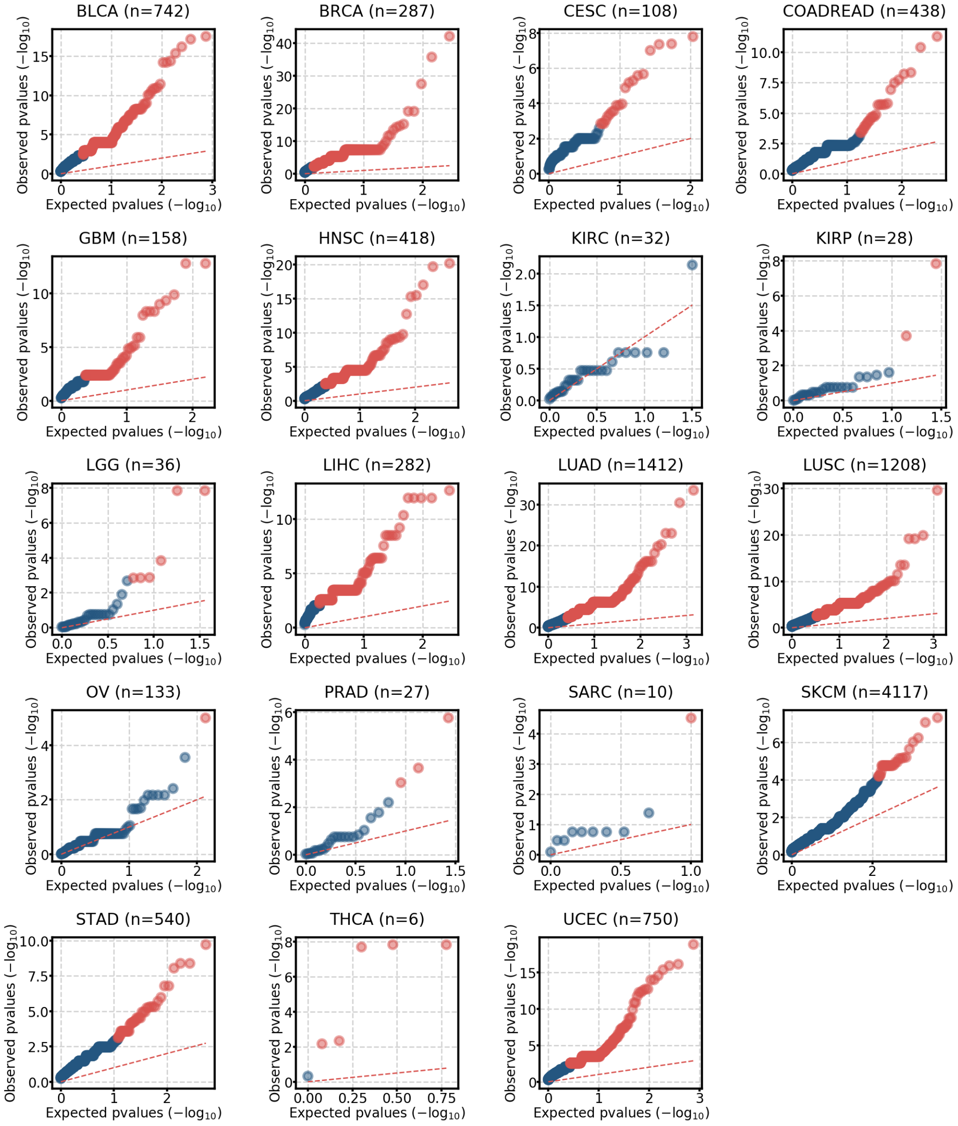

Supp. Fig. 4. Model adjustement by OncodriveCLUST. QQ-plots showing the observed p-values distribution versus the expected uniform distribution for all TCGA cohorts analyzed. Genes with q < 0.01 are highlighted in red. The number of p-values plotted on each QQ-plot is shown on top.

Supp. Fig. 5.

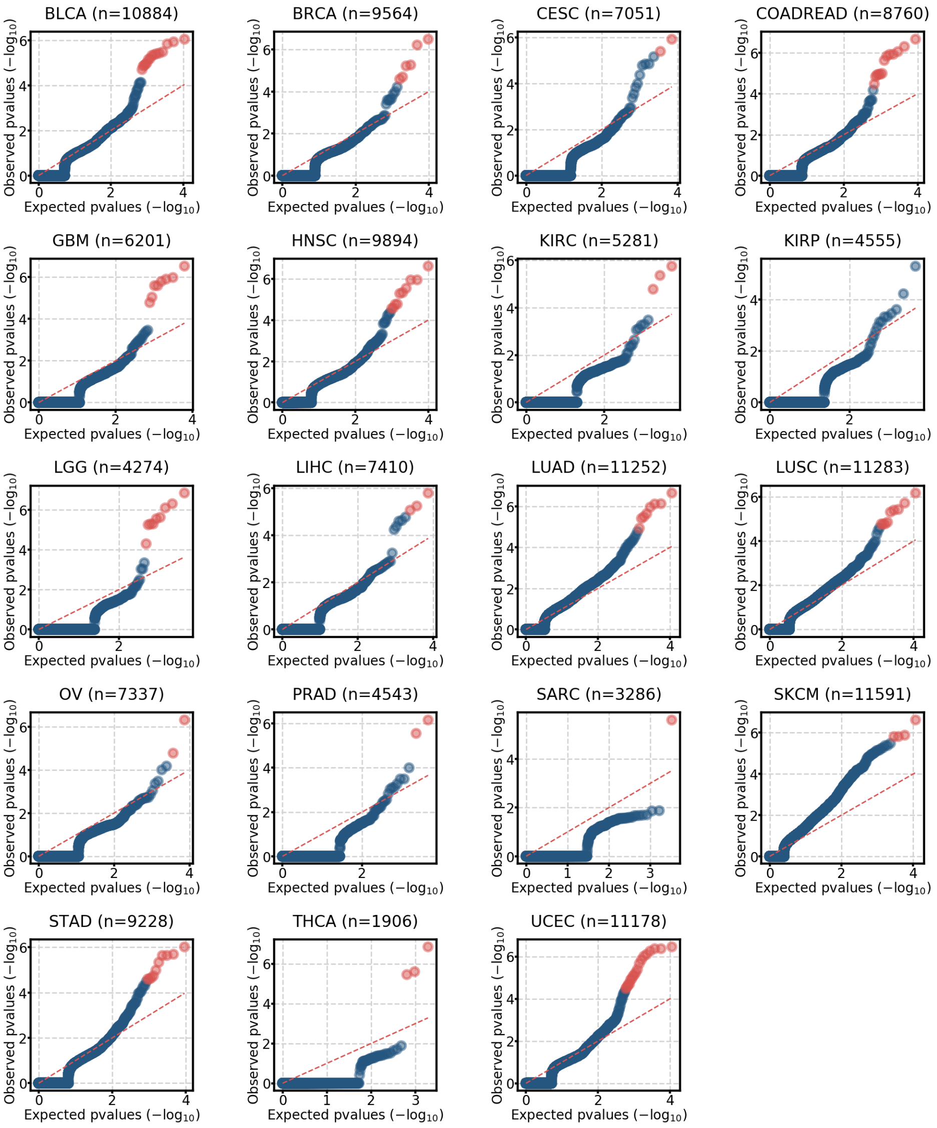

Supp. Fig. 5. Model adjustement by HotMAPS. QQ-plots showing the observed p-values distribution versus the expected uniform distribution for all TCGA cohorts analyzed. Genes with q < 0.01 are highlighted in red. The number of p-values plotted on each QQ-plot is shown on top.

Supp. Fig. 6.

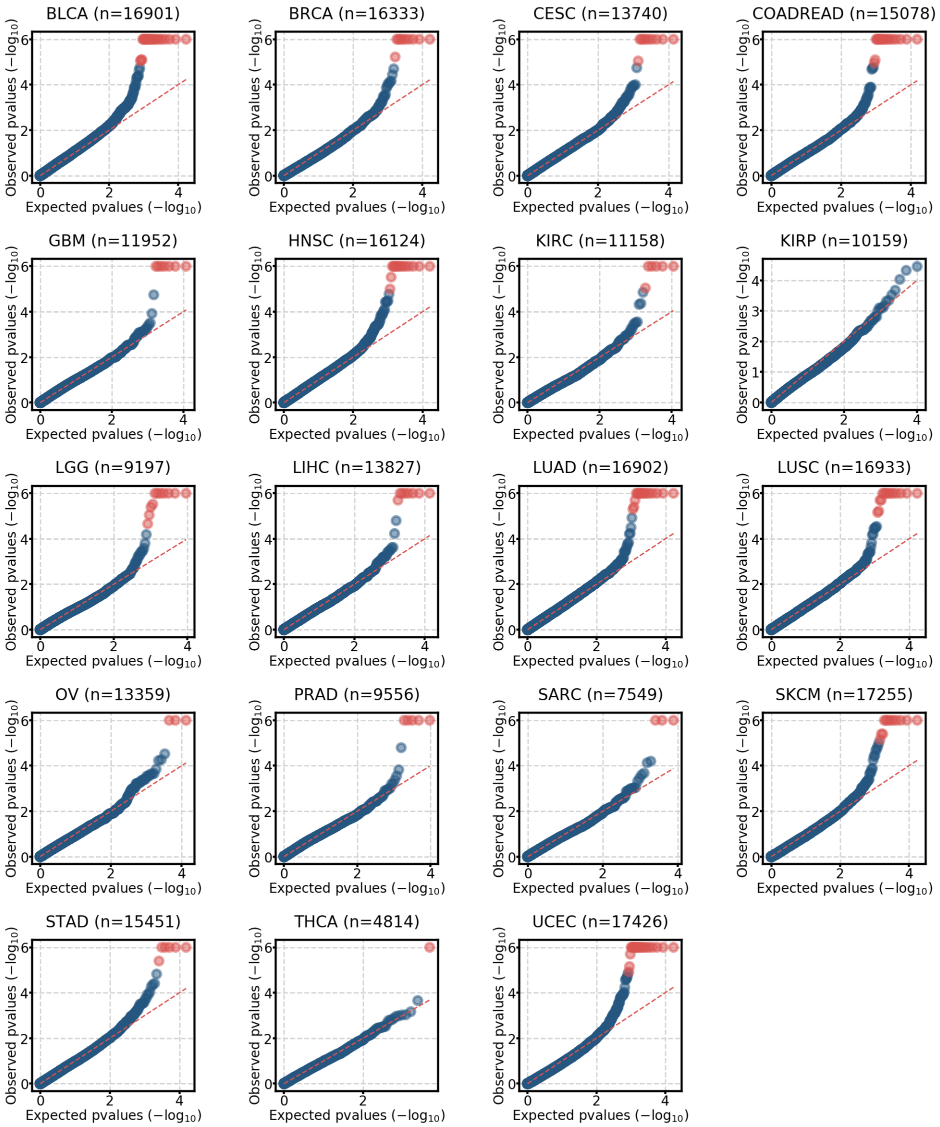

Supp. Fig. 6. Model adjustement by OncodriveFML. QQ-plots showing the observed p-values distribution versus the expected uniform distribution for all TCGA cohorts analyzed. Genes with q < 0.01 are highlighted in red. The number of p-values plotted on each QQ-plot is shown on top.

Supp. Fig. 7.

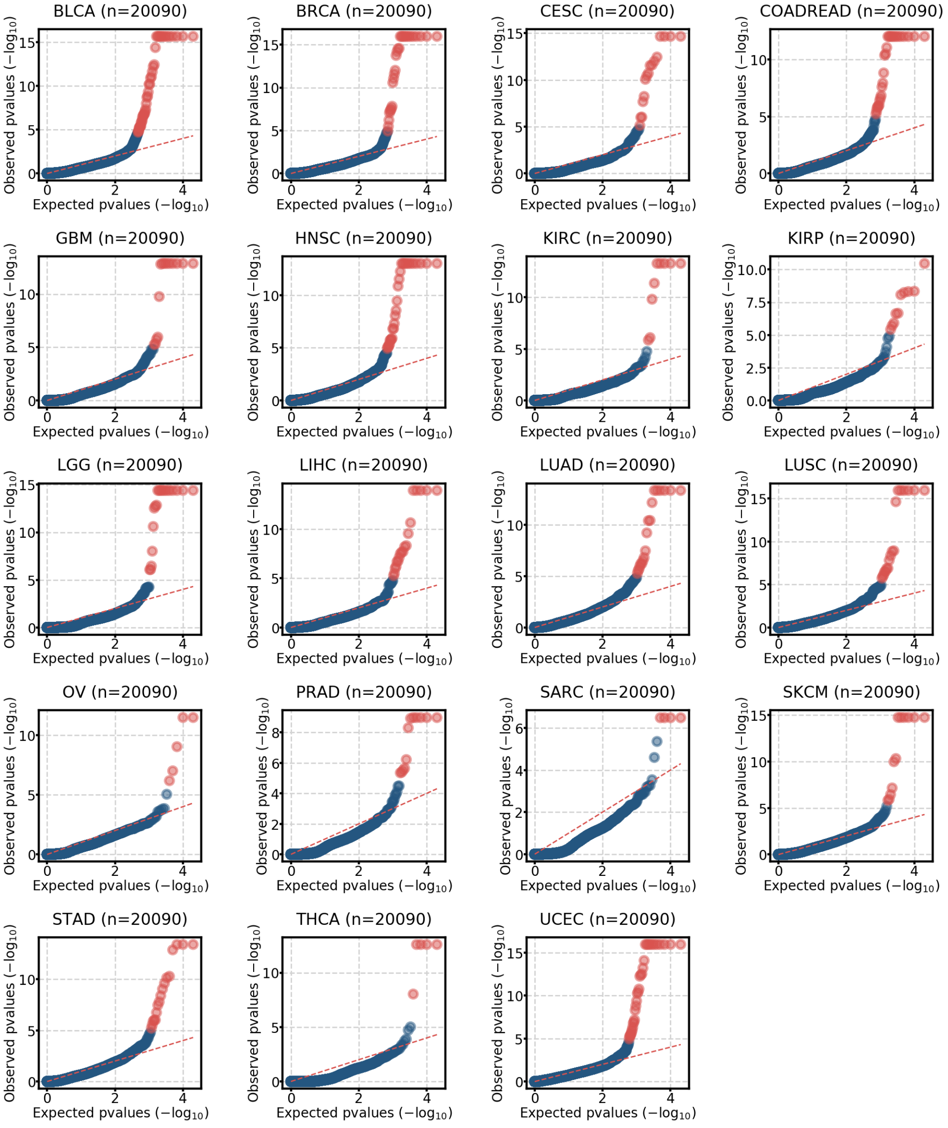

Supp. Fig. 7. Model adjustement by dNdScv. QQ-plots showing the observed p-values distribution versus the expected uniform distribution for all TCGA cohorts analyzed. Genes with q < 0.01 are highlighted in red. The number of p-values plotted on each QQ-plot is shown on top.

Supp. Fig. 8.

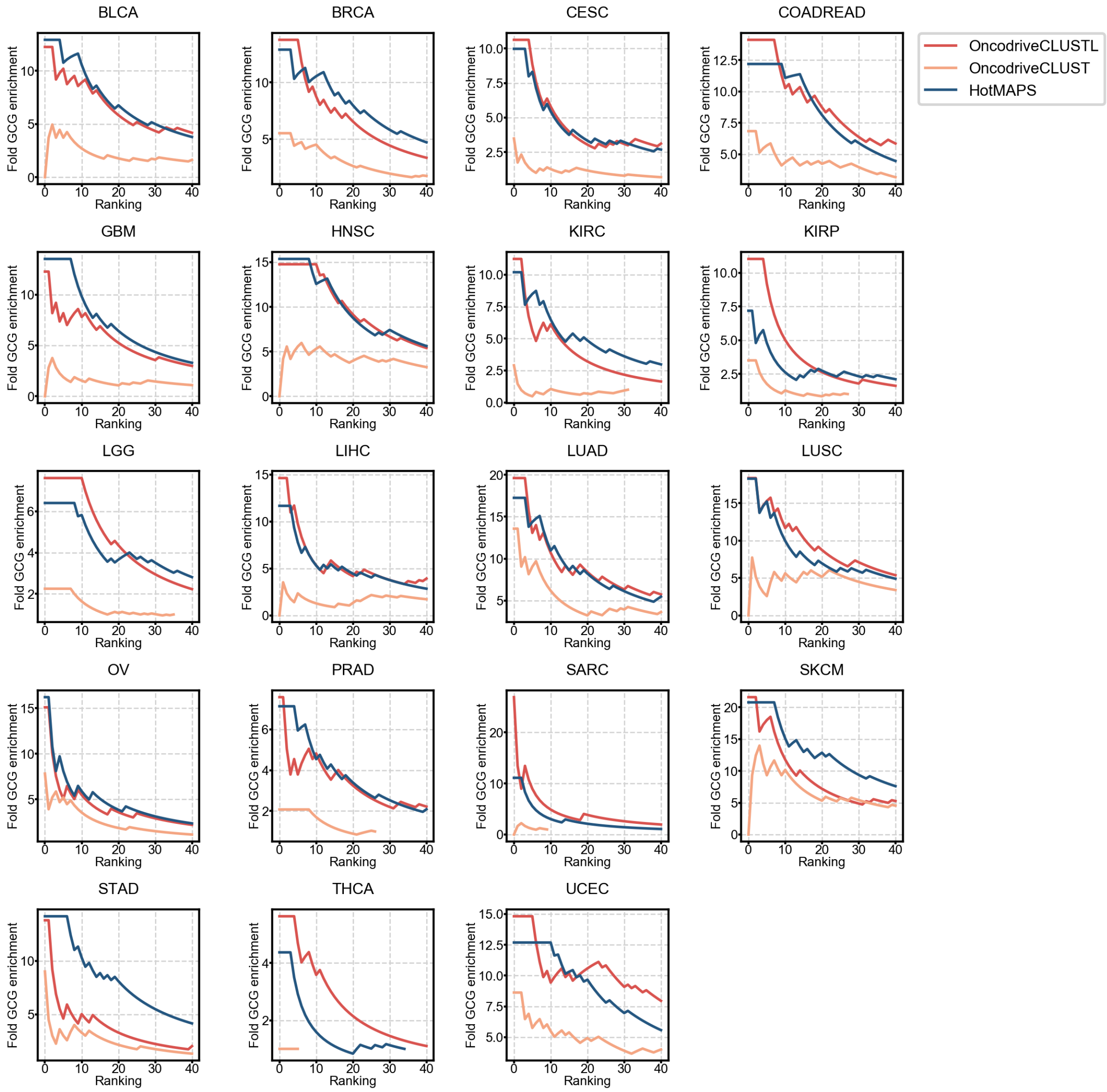

Supp. Fig. 8. Enrichment in CGC genes amongst OncodriveCLUSTL, OncodriveCLUST and HotMAPS top-ranking genes. Enrichment is computed as the fold increase in the proportion of CGC genes among sets with increasing number of the top 40 ranking genes.

Supp. Fig. 9.

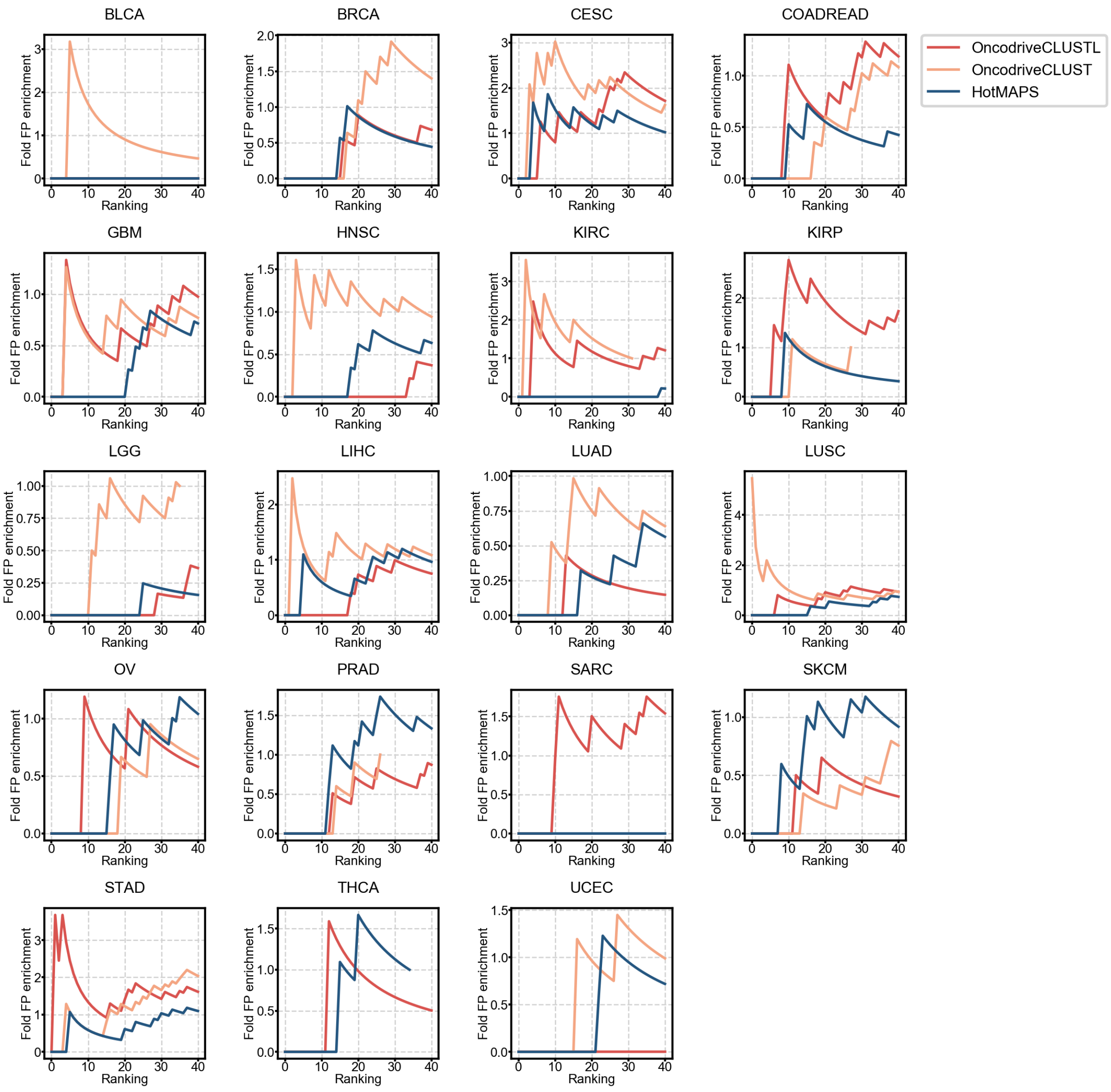

Supp. Fig. 9. Enrichment in false positive genes amongst OncodriveCLUSTL, OncodriveCLUST and HotMAPS top-ranking genes.E nrichment is computed as the fold increase in the proportion of false positive genes (FP) among sets with increasing number of the top 40 ranking genes detected by each method.

Supp. Fig. 10.

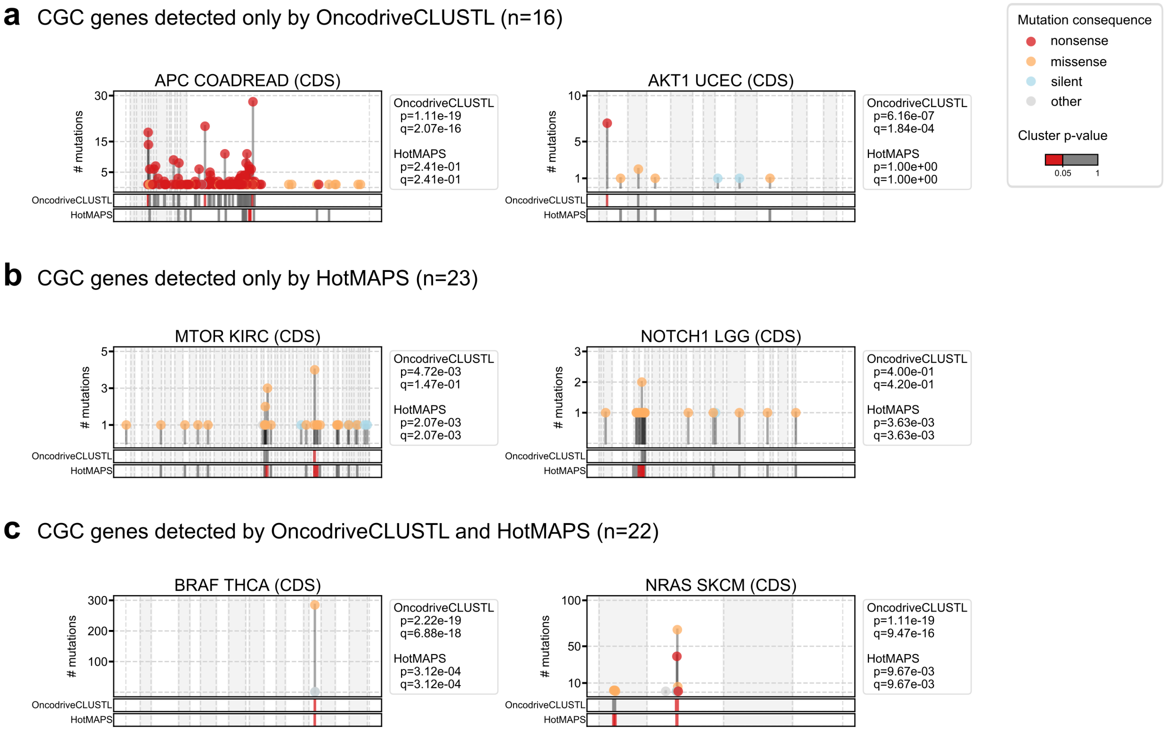

Supp. Fig. 10. Examples of CGC genes detected by OncodriveCLUSTL and HotMAPS. Two examples of CGC genes detected only by OncodriveCLUSTL (a), only by HotMAPS (b) or by both (c). For each example, the upper grid shows the total number of gene mutations labelled as nonsense (red), misense (orange), silent (blue) or other annotation (grey) mapped to the coding sequence of the gene. The middle grid shows clusters detected by OncodriveCLUSTL. Lower grid shows mutated residues mapped to nucleotide sequence detected by HotMAPS. Significant clusters or residues ($p<0.05$) are highlighted in red. Gene raw and adjusted p-values yielded by OncodriveCLUSTL and HotMAPS are shown for each example. OncodriveCLUSTL can detect clusters of nonsense mutations, which are skipped by the 3D analysis of HotMAPS (a). Although OncodriveCLUSTL can miss 3D clusters (b), its the linear clustering analysis can highlight the same mutated residues as HotMAPS (c). Check Supp. Benchmark section for further methodological details.

Supp. Fig. 11.

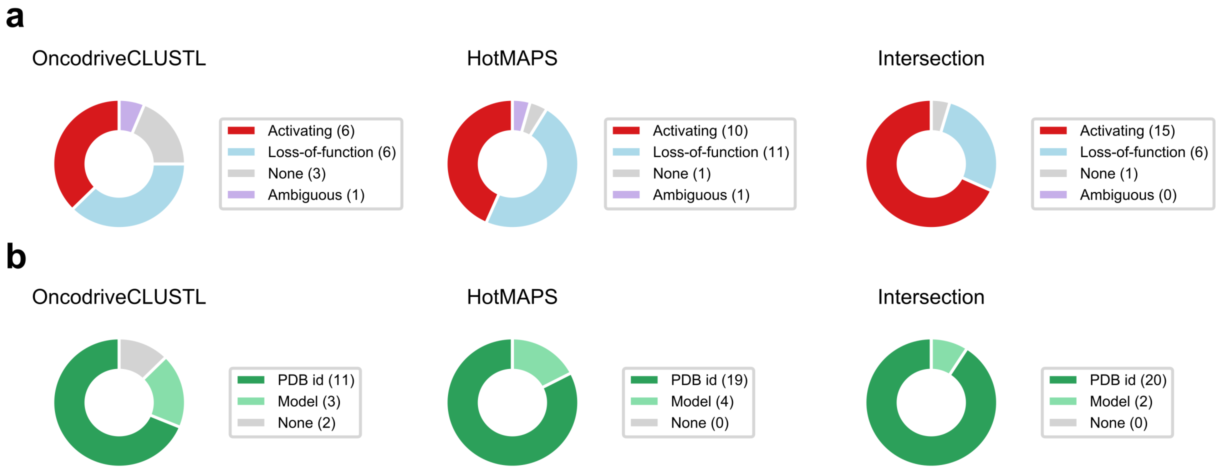

Supp. Fig. 11. Comparison of CGC genes detected by OncodriveCLUSTL and HotMAPS. Pie charts showing the proportion of CGC genes bearing activating, loss-of-function, ambiguous or no mode of action detected by OncodriveCLUSTL, HotMAPS, and both (a). Availability of PDB structure, 3D model or no protein structure of the CGC detected exclusively by OncodriveCLUSTL, HotMAPS, and both (b). Check Supp. Benchmark section for further methodological details.

Supp. Fig. 12.

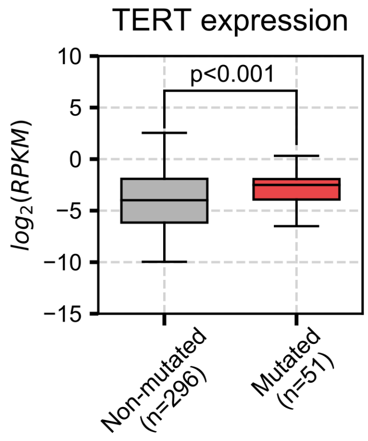

Supp. Fig. 12. TERT expression analysis. Samples containing a mutation in the chr5:1295228-1295253 cluster (red) detected by OncodriveCLUSTL have an increased TERT expression when compared to TERT promoter non-mutated samples (U-Mann Whitney $p < 0.001$). Only those samples with non-altered TERT gene copy number alterations were used for this analysis.

3 Supplementary Tables

Supp. Table 1. OncodriveCLUSTL running parameters for the tested cohorts. Best smoothing, clustering and simulation windows (bp) OncodriveCLUSTL according to the model selection strategy introduced in Supplementary Methods section (tabular file; 634 bytes).

Supp. Table 2. OncodriveCLUSTL results of genomic elements. Significant genomic elements ($q < 0.01$) found in the total 21 cohorts analyzed, including 19 TCGA WXS cohorts, Pancancer TCGA WGS-505 cohort and Mouse WXS dataset (tabular file; 15.5 kB).

Supp. Table 3. OncodriveCLUSTL clusters results. Clusters found in significant genomic elements for 19 TCGA WXS cohorts (hg19 coordinates), Pancancer TCGA WGS-505 cohort (hg19 coordinates) and Mouse WXS dataset (C3H_HeJ_v1.86 coordinates) (tabular file; 58.6 kB).

4 Supplementary references

Akrami,R. *et al.* (2013) Comprehensive Analysis of Long Non-Coding RNAs in Ovarian Cancer Reveals Global Patterns and Targeted DNA Amplification. *PLoS One*, **8**, e80306.

Alexandrov,L.B. *et al.* (2013) Signatures of mutational processes in human cancer. *Nature*, **500**, 415–421.

Connor,F. *et al.* (2018) Mutational landscape of a chemically-induced mouse model of liver cancer. *J. Hepatol.*, **69**, 840–850.

Ellrott,K. *et al.* (2018) Scalable Open Science Approach for Mutation Calling of Tumor Exomes Using Multiple Genomic Pipelines. *Cell Syst.*, **6**, 271–281.e7.

Fredriksson,N.J. *et al.* (2014) Systematic analysis of noncoding somatic mutations and gene expression alterations across 14 tumor types. *Nat. Genet.*, **46**, 1258–1263.

Frigola,J. *et al.* (2017) Reduced mutation rate in exons due to differential mismatch repair. *Nat. Genet.*, **49**, 1684–1692.

Harrow,J. *et al.* (2012) GENCODE: the reference human genome annotation for The ENCODE Project. *Genome Res.*, **22**, 1760–74.

Lawrence,M.S. *et al.* (2013) Mutational heterogeneity in cancer and the search for new cancer-associated genes. *Nature*, **499**, 214–218.

Martincorena,I. *et al.* (2017) Universal Patterns of Selection in Cancer and Somatic Tissues. *Cell*, **171**, 1029–1041.e21.

McLaren,W. *et al.* (2016) The Ensembl Variant Effect Predictor. *Genome Biol.*, **17**, 122.

Mularoni,L. *et al.* (2016) OncodriveFML: A general framework to identify coding and non-coding regions with cancer driver mutations. *Genome Biol.*, **17**, 128.

Przytycki,P.F. and Singh,M. (2017) Differential analysis between somatic mutation and germline variation profiles reveals cancer-related genes. *Genome Med.*, **9**, 79.

Safran,M. *et al.* (2003) Human Gene-Centric Databases at the Weizmann Institute of Science: GeneCards, UDB, CroW 21 and HORDE. *Nucleic Acids Res.*, **31**, 142–146.

Sondka,Z. *et al.* (2018) The COSMIC Cancer Gene Census: describing genetic dysfunction across all human cancers. *Nat. Rev. Cancer*, 1.

Tamborero,D. *et al.* (2018) Cancer Genome Interpreter annotates the biological and clinical relevance of tumor alterations. *Genome Med.*, **10**, 25.

Tamborero,D. *et al.* (2013) OncodriveCLUST: Exploiting the positional clustering of somatic mutations to identify cancer genes. *Bioinformatics*, **29**, 2238–2244.

Tokheim,C. *et al.* (2016) Exome-scale discovery of hotspot mutation regions in human cancer using 3D protein structure. *Cancer Res.*, **76**, 3719–3731.
